# Supplementary material for: Time-series transcriptome analysis identified differentially expressed genes in broiler chicken infected with mixed Eimeria species
Source: Front Genet. 2022 Aug 8;13:886781. doi: 10.3389/fgene.2022.886781 (PMC9393255; doi:10.3389/fgene.2022.886781)
Supplement: Supplementary file 2 [file DataSheet1.ZIP › 4dpi_KEGG.Gsea.1625100298584/KEGG_LIMONENE_AND_PINENE_DEGRADATION.html]

Details for gene set KEGG\_LIMONENE\_AND\_PINENE\_DEGRADATION[GSEA]

|  || Dataset | TMM\_4dpi\_gct\_format\_4dpi\_gct\_format.Class\_4dpi.cls #PC\_versus\_NC.Class\_4dpi.cls #PC\_versus\_NC\_repos |
| Phenotype | Class\_4dpi.cls#PC\_versus\_NC\_repos |
| Upregulated in class | 0 |
| GeneSet | KEGG\_LIMONENE\_AND\_PINENE\_DEGRADATION |
| Enrichment Score (ES) | -0.71729106 |
| Normalized Enrichment Score (NES) | -1.5684906 |
| Nominal p-value | 0.038971808 |
| FDR q-value | 0.19797532 |
| FWER p-Value | 0.8068 |
Table: GSEA Results Summary

  

Fig 1: Enrichment plot: KEGG\_LIMONENE\_AND\_PINENE\_DEGRADATION      
 Profile of the Running ES Score & Positions of GeneSet Members on the Rank Ordered List

  

| SYMBOL | TITLE | RANK IN GENE LIST | RANK METRIC SCORE | RUNNING ES | CORE ENRICHMENT || 1 | YOD1 | na | 4282 | 0.143 | -0.3005 | No |
| 2 | ALDH3A2 | na | 5110 | 0.069 | -0.3422 | No |
| 3 | HADHA | na | 7423 | -0.129 | -0.4844 | No |
| 4 | ALDH7A1 | na | 10220 | -0.448 | -0.5418 | Yes |
| 5 | ALDH9A1 | na | 10288 | -0.458 | -0.3680 | Yes |
| 6 | EHHADH | na | 10699 | -0.538 | -0.1913 | Yes |
| 7 | ECHS1 | na | 11432 | -0.767 | 0.0482 | Yes |
Table: GSEA details [plain text format]

  

Fig 2: KEGG\_LIMONENE\_AND\_PINENE\_DEGRADATION      
 Blue-Pink O' Gram in the Space of the Analyzed GeneSet

  

Fig 3: KEGG\_LIMONENE\_AND\_PINENE\_DEGRADATION: Random ES distribution      
 Gene set null distribution of ES for **KEGG\_LIMONENE\_AND\_PINENE\_DEGRADATION**
